# The Role of the CLIC/GEEC Endocytic Pathway for Mechanophysical Transfection of DNA

**DOI:** 10.1002/jgm.70059

**Published:** 2025-11-26

**Authors:** Sean Weaver, Emran O. Lallow, Kelly Kyker‐Snowman, Nandita C. Jhumur, Melissa Gulley, Christine C. Roberts, David I. Shreiber, Hao Lin, Joel N. Maslow

**Affiliations:** ^1^ GeneOne Life Science, Inc. Seoul Republic of Korea; ^2^ Department of Mechanical Engineering and Aerospace Engineering, Rutgers The State University of New Jersey Piscataway New Jersey USA; ^3^ Department of Biomedical Engineering, Rutgers The State University of New Jersey Piscataway New Jersey USA; ^4^ Department of Medicine Morristown Medical Center Morristown New Jersey USA; ^5^ Department of Cardiovascular Sciences, Houston Methodist Weill Cornell School of Medicine Houston Texas USA

## Abstract

The clathrin‐independent carriers/glycosylphosphatidylinositol‐attached protein‐enriched endosomal compartments (CLIC/GEEC) pathway is a clarithin‐ and dynamin‐independent endocytic pathway. CLIC/GEEC‐mediated endocytosis is a large‐capacity, rapid‐acting process that is utilized by cells for bulk uptake of nutrients and membrane‐anchored cargo and for homeostatic maintenance of membrane tension. Here, we present a brief overview of this pathway, including its known molecular mechanisms, inhibitors, activity in different cell types, and its potential relevance in mechanophysical DNA transfection.

## Graphical abstract

The clathrin‐independent carriers/GPI‐enriched endocytic compartments (CLIC/GEEC or CG) pathway is a high‐capacity, clathrin‐, and dynamin‐independent route for nutrient and membrane‐anchored cargo uptake of DNA. This overview highlights its molecular mechanisms, regulation, cellular roles, and relevance to mechanophysical DNA transfection.

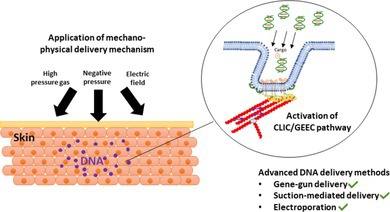

**Abbreviations**
ARF1: ADP‐ribosylation factor 1
Arp2/3: Actin‐related protein 2/3
BDP: BAR domain protein
CHO: Chinese hamster ovary
CIE: clathrin‐independent endocytosis
CLIC/GEEC: clathrin‐independent carriers/glycosylphosphatidylinositol‐attached protein‐enriched endosomal compartments
CLICs: clathrin‐independent carriers
CME: caveola‐mediated endocytosis
CME: clathrin‐mediated endocytosis
CTxB: cholera toxin B
EP: electroporation
eTE: electrotransfection efficiency
FAS: focal adhesion sites
GAP: GTPase activating protein
GPI‐AP: glycosylphosphatidylinositol‐anchored protein
IRSp53: insulin receptor substrate protein of 53 kDa
MEFs: mouse embryonic fibroblasts
MOA: mechanism of action
NFIS: needle‐free injection system
pDNA: plasmid DNA
PM: plasma membrane
TfR: transferrin receptor

## 1 Introduction

Endocytosis is a key cellular process involved in nutrient uptake, homeostasis, and migration. This mechanism has been leveraged for DNA uptake for the purpose of exogenous gene expression, genetic editing, and chromosomal mutation [1, 2, 3]. The two best characterized endocytic mechanisms to date are clathrin‐ and caveolae‐mediated endocytosis. During clathrin‐mediated endocytosis (CME), clathrin forms a triskelion “cage” around clathrin‐coated pits in the plasma membrane (PM) of a cell to facilitate uptake and create a capsule for further transport within the cell [4]. In caveolae‐mediated endocytosis, the membrane protein caveolin pools in domains enriched with cholesterol and sphingolipids, which results in a flask‐shaped invagination that is released into the intracellular space [5]. While caveolae‐mediated endocytosis does not require clathrin, both pathways are dependent on the scission protein dynamin to bud the developed invaginations into the cell.

On the other hand, various clathrin‐ and caveolae‐independent endocytic processes have also been identified [1, 6, 7, 8]. These include the clathrin‐independent carriers/glycosylphosphatidylinositol‐attached protein‐enriched endosomal compartments (CLIC/GEEC) endocytosis pathway, fast endophilin‐mediated endocytosis, flotillin‐mediated endocytosis, macropinocytosis, and phagocytosis [4, 8, 9, 10, 11, 12, 13, 14]. Each of these is triggered by varying mechanisms and has a wide range of signaling and effector molecules involved in their execution. These pathways can be subcategorized based on whether or not they require the scission protein dynamin or utilize an alternative mechanism to separate the invaginated endocytic tubule from the cell's PM to create “free” vesicles [15].

**FIGURE 1:**
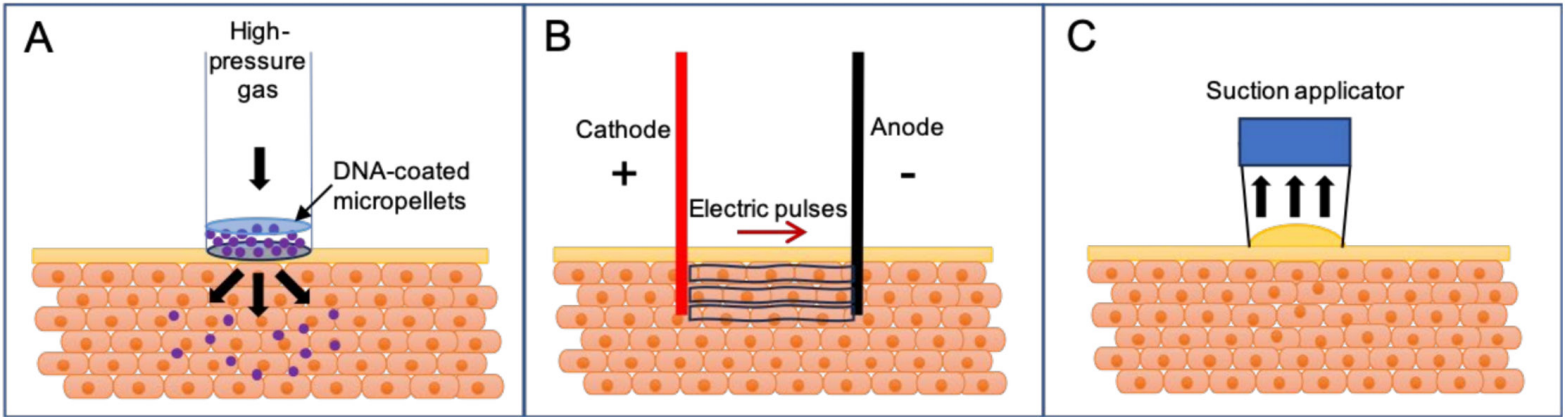
Schematics of mechanophysical in vivo DNA transfection techniques. (A) Microjet impingement via a needle‐free system, where DNA‐loaded microdroplets penetrate tissue under high speed. (B) In EP the applications of electric fields are necessary to trigger endocytosis. (C) Cutaneous suction causes tissue deformation‐relaxation and leads to DNA transfection.

This review is motivated by interests in potential pathways that mediate DNA transfection in vivo, especially with physical means, such as suction‐ and electroporation‐mediated DNA uptake [16, 17, 18]. A clear understanding of how mechanotransduction contributes to the mechanism of action (MOA) of plasmid uptake provides opportunities to optimize transfection and subsequent protein expression of encoded transgenes, which is in turn crucial to eliciting stronger and/or more rapid therapeutic responses. While mRNA vaccines are currently transfected into cells via a lipid nanoparticle capsule [4], delivery of plasmid DNA (pDNA) requires physical methods to enhance the efficiency of cellular uptake, with mechanophysical or electrical methods as the most commonly used in clinical practice. Currently, mechanophysical methods to induce in vivo pDNA transfection include forced delivery of microdroplets by a needle‐free injection system (NFIS) [19, 20], electroporation (EP) [21, 22, 23, 24, 25, 26], and suction [16, 27] (Figure 1). In EP, the application of an electric field local to the delivery site for pDNA is used to induce cellular uptake and transfection within both in vitro and in vivo settings [12, 28, 29, 30]. NFIS utilizes high pressure for the ballistic delivery of pDNA microdroplets to a precise tissue region.

Different from these previously explored options, our group has demonstrated that the suction‐mediated in vivo DNA transfection method is a highly effective method to induce cellular uptake of pDNA. Suction, when applied to the skin locally to where pDNA has been injected intradermally, results in efficient pDNA uptake and expression within 1 h and yields immune responses at least 100‐fold greater than the injection of pDNA alone [16]. Moreover, suction‐mediated in vivo transfection in rats was shown to induce equivalent B cell responses but superior T cell immune responses relative to either electroporation or NFIS‐mediated delivery of a COVID‐19 DNA vaccine [27]. Clinical trial data have also demonstrated that suction‐mediated transfection of COVID‐19 DNA vaccines yielded similar B cell responses, but approximately 20‐fold greater T cell responses, relative to either EP‐ or NFIS‐mediated vaccine delivery [31, 32] Overall suction‐mediated in vivo transfection has been demonstrated as effective in rats, hamsters, rabbits, and human clinical trials [27, 31, 33]. The mechanism of pDNA transfection induced by these mechanophysical stimuli remains, however, to be fully characterized.

In this review we focus on the CLIC/GEEC pathway, as it is a strong candidate in both mechanical‐ and EP‐mediated transfection. Here, we review the molecular process of CLIC/GEEC endocytosis, its cell‐type dependence and inhibitors, discuss its possible relevance in the uptake of pDNA in vitro and in vivo, and propose possible future studies to validate these findings.

## 2 THE Process of CLIC/GEEC‐Mediated Endocytosis

The CLIC/GEEC pathway is both a clathrin‐ and dynamin‐independent mechanism of endocytosis [34]; particles can be internalized from the extracellular space without the assistance of the scaffolding protein clathrin or the scission protein dynamin. This process generally functions as one of the endocytic pathways responsible for rapid fluid uptake and has been implicated in glycosylphosphatidylinositol‐anchored protein (GPI‐AP) internalization, as well as cellular uptake of some toxins such as cholera toxin B (CTxB) and Shiga toxin [4]. CLIC/GEEC‐mediated endocytosis is thought to be fast acting in response to changes in tension in the cell's PM [35]. The process begins with tubule‐shaped invaginations attached to the PM, which develop into clathrin‐independent carriers (CLICs) that later mature into GPI‐AP enriched early endosomal compartments (GEECs). Once internalized and budded from the PM within the cell, GEECs are either broken down and recycled to return surface receptors back to the membrane, or transported to the lysosomes for degradation [3]. CLIC/GEEC‐mediated uptake can be divided into three steps: (1) activation, (2) tubule elongation, and (3) vesicle scission [1, 7, 14, 15]. Key molecules involved are summarized in Table 1.

**TABLE 1:** List of molecules involved and their functions pertinent to the CLIC/GEEC endocytic pathway.

	Molecule	Description	Major function	References
Mechanosensing and activation	Vinculin	Integrin‐ and talin‐bound cytoskeletal protein	Transduces PM tension changes	[7]
	Talin	Integrin bound cytoskeletal protein	Transduces PM tension changes	[7]
	GBF1	GEF for Arf1	Stimulates dissociation of Arf1 and GDP	[15]
	Arf1	ARF family GTPase	Recruits ARHGAP10 which assists in maintaining downstream dynamic actin polymerization and limits PICK1 activity	[15]
Tubule elongation	PICK1	BAR domain‐containing Protein	Temporarily holds on activation of Arp2/3	[15]
	Arp2/3	Actin regulating protein	Stimulates polymerization and eventual membrane nucleation of F‐actin	[15]
	Cdc42	Rho GTPase	Activates BDPs and Arp2/3	[7, 15]
Vesicle scission	IRSp53	I‐BAR domain‐containing Protein	Senses and accentuates negative curvature in the PM; Assists in vesicle scission	[15]
	GRAF1*	BAR domain‐containing Protein	Senses and accentuates positive curvature in the PM; Assists in vesicle scission	[1, 36, 37]
General	Cholesterol	Sterol lipid present in animal cell PMs	Facilitates changes in PM properties and most endocytic pathways	[4, 38, 39]
	PtdIns(4,5)P2	Phospholipid PM component	Substrate for signaling proteins	

*Note:* 1* Indicates conflicting evidence.

### 2.1 Activation

**FIGURE 2:**
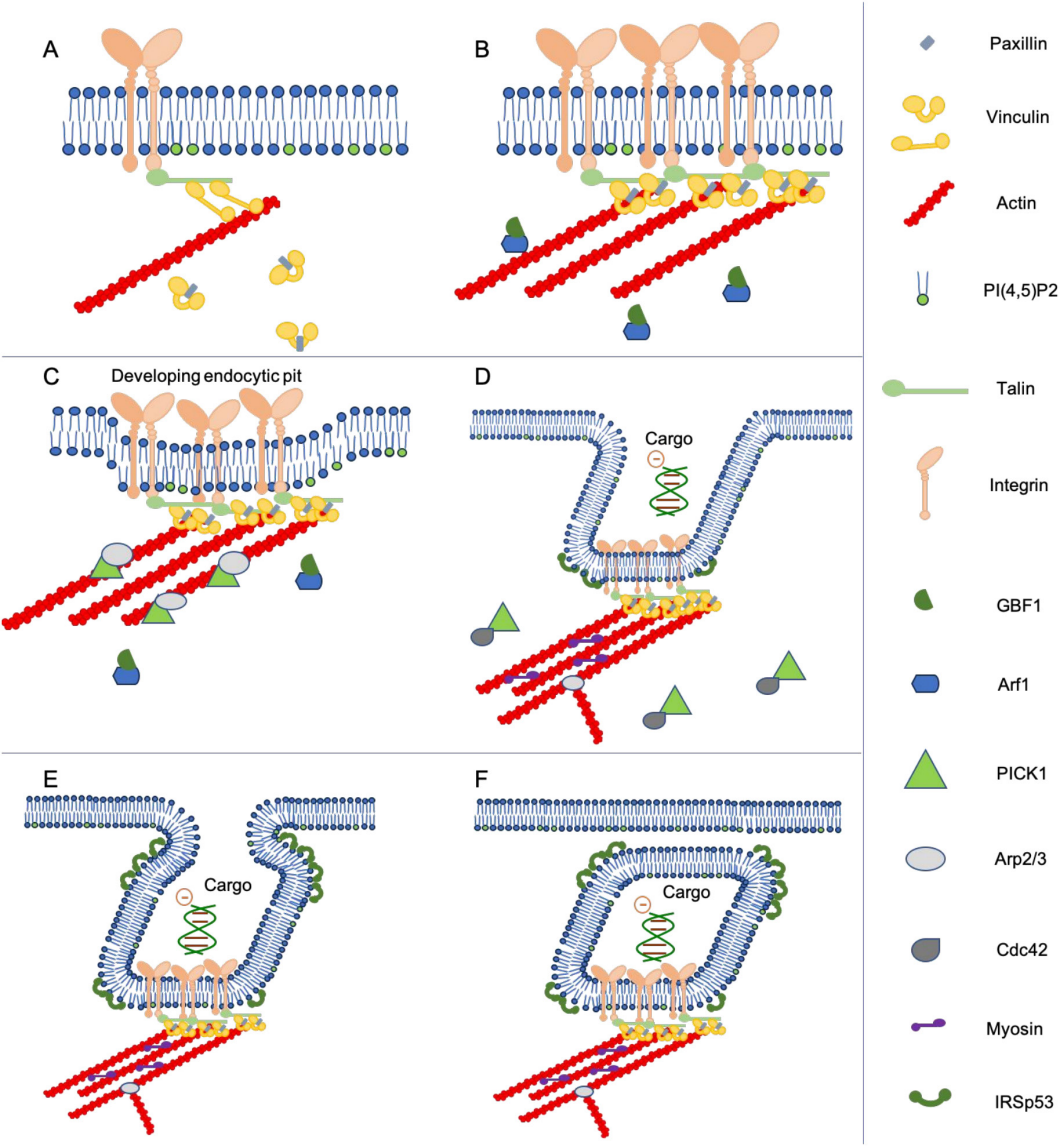
Molecules and pathways for activation. (A) Focal tension activates vinculin through talin stretching, which in turn inhibits localization of GBF1 and Arf1 and downregulates actin activity to ameliorate added PM tension. (B) Tension release deactivates vinculin, which allows rapid localization of GBF1 to the PM at the FAS that in turn assists Arf1 in its stimulation to release GDP in preparation to be reloaded with GTP. (C) GBF1 and Arp2/3 are recruited near a forming endocytotic pit; Arp2/3 activity is temporarily on hold by PICK1 to allow further pit development. (D) Cdc42 arrives at the FAS and disperses PICK1 in conjunction with Arf1, allowing Arp2/3 to spontaneously activate, leading to a multitude of actin monomers to polymerize into F‐Actin fibrils that pull the endocytic pit. IRSp53 also localizes to enhance the negative curvature and shape the pit into a tubule. (E) IRSp53 collects at the neck of the tubule against the PM and begins to pinch it in towards itself to create a friction reaction and bud the tubule from the PM altogether. (F) The tubule is free from the PM and is subject to the cellular recycling mechanisms.

When mechanical stress is applied to a cell, whether by osmotic changes, bulk force pressure, or direct stretching, tension, and strain are increased at the PM. Initially, preexisting invaginations and ruffles in the PM will smooth out in response to applied external tension, similar to slack in a rope. Once this buffering mechanism has been exhausted, the membrane is stretched, and resting tension is increased proportional to the force applied. This stress is especially experienced at focal adhesion sites (FAS) where transmembrane integrin receptors protrude into the extracellular space and connect to the intracellular cytoskeleton protein, talin (Figure 2A) [7]. Talin is connected to the actomyosin skeleton of the cell by other signaling molecules that allow it to transduce mechanical feedback from the exterior and trigger mechanobiological reactions within the cell [36]. The most notable signaling molecule is the transduction protein vinculin, which plays a significant role in the mechanosensitivity of cells and their responses [7]. As stress transmits through the integrin, the connected talin molecule is physically elongated. This causes the talin‐attached vinculin molecules to convert to their active form. This process inhibits the localization of GBF1, a GTP exchange factor for the CLIC/GEEC‐GTPase ADP‐ribosylation factor 1 (ARF1), onto the cell membrane (Figure 2A) [7]. When activated, ARF1 initiates the recruitment of ARHGAP10, the RhoGAP for CDC42, which assists in maintaining the dynamic actin polymerization process responsible for moderating the cellular membrane tension [7]. Thus, when strain is applied, activated vinculin inhibits GBF1 localization, resulting in reduced downstream actin activity and slowing processes that would increase cellular tension, such as endocytosis. Once the stress on the cell is removed and the strain is relaxed, PM tension rapidly decreases. Vinculin is deactivated, which triggers the rapid localization of GBF1 to the PM at the FAS (Figure 2B). As the GEF for Arf1, GBF1 facilitates its release of GDP, thus increasing GTP turnover and in turn the rate of endocytosis [37]. In upregulating endocytosis in this way after applied stress is removed, a cell is actively preserving membrane area homeostasis and ensuring that it does not experience membrane damage due to large changes in environmental pressure.

### 2.2 Tubule Elongation

Upon relaxation of the applied stress/strain, recruitment of GBF1 and Arf1 to the FAS initiates the formation of an endocytic pit (Figure 2C) [15]. Actin‐related protein 2/3 (Arp2/3), which nucleates the polymerization of local actin monomers, is also recruited at this time, but is held in an inactive state by the BAR domain protein (BDP) interacting with PRKCA 1 (PICK1) [15]. This is evidenced by the lack of actin polymerization until the dispersion of PICK1, which coincides with the arrival of another GTPase, Cdc42, from the PM, as depicted in Figure 2D. Preventing premature actin polymerization allows the initial pit to continue to develop until the localization of Cdc42. The latter event not only suppresses PICK1 and allows for the commencement of actin polymerization via the activation of Arp 2/3 but also activates another BDP, insulin receptor substrate protein of 53 kDa (IRSp53) [6, 15]. IRSp53 has an I‐BAR domain that has a convexly curved structure that is both negative curvature sensing and inducing [15]. This allows it to sense the developing fold in the PM, localize to the area, and bind to the membrane to induce more curvature relative to its concentration. Such action helps create a tubular sculpting scaffolding from the early invagination and further develops it to a CLIC. Meanwhile, the freshly polymerized actin fibers nucleate at the intracellular membrane and utilize pulling force from the actin cytoskeleton and myosin II motors to elongate the CLIC. This force is suspected to be the driving force shaping the elongated tubule [3] (Figure 2D).

It is worth noting that other studies have instead suggested that the GTPase activating protein (GAP) from the Rho GTPase subfamily GRAF is responsible for the curvature of the endocytic pit and the creation of the tubule through its BAR and PH domains in the form of a GTPase regulator associated with focal adhesion kinase 1 (GRAF1) [1, 13, 40, 41]. The BAR and PH domains contained in GRAF1 have been shown to cause the localization of GRAF1 to the PtdIns(4,5)P2‐enriched regions of the PM and the tubularly shaped regions of lipid structures [42, 43]. Thus, GRAF1 has been suggested to be an essential component of CLIC/GEEC processes [1, 13, 40, 41, 44, 45]. Nevertheless, there is also strong speculation deviating from the above hypothesis, that GRAF1 may be a central component in alternative means of membrane tension control that merely resemble or work simultaneously with CLIC/GEEC in response to mechanochemical stimulation, independent of the CLIC/GEEC pathway [7]. These aspects will be further discussed below in the specific case of HeLa cells.

We also note that more remains to be learned regarding the precise location of tubule formation and elongation. The GLectin hypothesis, which suggests that local membrane lipid concentrations ideal for deep PM invaginations promote ensuing tubule formation, offers one possible mechanism [6, 13, 14]. This hypothesis proposes that membrane‐bound glycosylated cargo localizes monomeric Gal‐3 to the PM where it oligomerizes with other local Gal‐3 monomers. The induced clustering of cargo proteins and glycosphingolipids creates a strain on the PM that leads to membrane buckling and the eventual development of a CLIC. Although GLectin‐mediated tubule formation has been observed to be in sync with CLIC/GEEC endocytosis, it has not yet been determined whether it is a universal component of the uptake method or just one of many mechanisms to invoke membrane bending.

### 2.3 Vesicle Scission

The process causing the release of the PM‐connected tubule into the intracellular space as a free vesicle remains poorly understood. Unlike most well‐described methods of endocytosis, the CLIC/GEEC pathway has been shown to be, for the most part, completely uninhibited in cell cultures with a triple knockdown of the scission protein dynamin [7]. Though some studies dispute the assertion that CLIC/GEEC is completely void of dynamin [1, 36], it is widely accepted that the CLIC/GEEC pathway does not utilize dynamin as the main method of scission, unlike CME and caveolae‐dependent endocytosis [7, 13, 14, 34, 41].

There are a few schools of thought on the mechanisms of vesicle scission in CLIC/GEEC endocytosis. The most common hypothesis is that one or both of the BDP IRSp53, or GRAF1 coat the entire vesicle and enrich specifically at the neck against the membrane, creating a “scission scaffolding” that constricts over time [1, 3, 15] (Figure 2E). Once the PM narrows sufficiently, it may undergo fission via a friction‐based reaction [6, 14, 15]. Tautness of the PM at the neck is exacerbated by the ratcheting action of the actin cytoskeleton as well, assisting in the friction‐based scission [3].

While the above process offers a reasonable description of vesicle scission from the PM, this aspect of CLIC/GEEC endocytosis is still very much a current subject of debate [1, 7, 15, 36], and the definitive events determining vesicle budding remain to be confirmed. For example, Thottacherry et al. suggest an alternative hypothesis where EndoA‐coated tubules drive scission by creating friction between the tubule‐forming scaffolds and the PM itself [14]. Other studies argue that, while CLIC/GEEC endocytosis and other clathrin‐independent endocytosis (CIE) in general are largely able to function without dynamin, this scission protein may still work with the BDP to constrict the neck of a budding vesicle [1].

Once a vesicle is released from the PM, it migrates within the cell via the actin cytoskeleton [3, 14], as depicted in Figure 2F, and is subjected to the cell's general sorting mechanisms, followed by either the vesicle's deconstruction or recycling by the cell's lysosomes or late endosomes [14, 46].

## 3 Inhibitors of the CLIC/GEEC Pathway

As this review is motivated by the possible relevance of the CLIC/GEEC pathway in mechanic‐physical transfection methods, means to effectively interrogate its involvement (or absence) during the processes are necessary and important. A common approach is to use inhibitors that target the various steps within the pathway. We summarize these inhibitors or inhibiting factors in this section. This knowledge is also helpful in designing future experiments as discussed in section 6. Currently known inhibitors and their respective functions are listed in Table 2; for each, we also discuss their specificity.

**TABLE 2:** Inhibitors, respective function, and specificity to CLIC/GEEC.

Inhibitors	Function	References
Specific		
LG186	Inhibits GBF1, which subsequently disrupts the ability of Arf1 to hydrolyze GTP in an efficient manner, therefore disrupting CLIC/GEEC endocytosis at the activation step	[4, 7, 15]
Arf1‐specific shRNA	Inhibits the RhoGTPase Arf1 from performing its function in the CLIC/GEEC pathway, not allowing for the suppression of PICK1, nor the hydrolysis of GTP; disrupts both activation and elongation	[34]
7‐Ketocholesterol	Reduces membrane order by preventing close packing of acyl chains	[4, 38, 39]
FSC231	Inhibits PICK1 PDZ domain which inhibits CLIC/GEEC fluid‐phase endocytosis in a dose‐dependent manner; often used for neuroscience applications	[15]
PICK1 knockout cells	Similar to FSC231	[15]
IRSp53 knockout cells	IRSp53 depletion disrupts actin dynamics and vesicle sculpting.	[15]
Partially specific		
Low temperature (< 25°C)	Lower temperature inhibits CLIC/GEEC much more dramatically when compared with other CME and CIE pathways.	[4, 7]
GRAF1‐specific siRNA	A possible inhibitor of CLIC generation and GEEC internalization	[1, 36]
ML141	An allosteric inhibitor of the RhoGTPase Cdc42, an essential component of the CLIC/GEEC pathway. Also effective in inhibiting macropinocytosis [47]	[7]
Nonspecific		
CK666	Inhibits actin assembly by stabilizing the inactive form of the Arp2/3 complex, which in turn inhibits several forms of endocytosis that rely on actin polymerization. A dose‐dependent inhibitor	[15, 48]
Latrunculin	An actin polymerization inhibitor that affects all general forms of endocytosis that require dynamic actin	[15, 49]
Jasplakinolide	An actin filament stabilization inhibitor/actin polymerization inducer that affects all general forms of endocytosis that requires dynamic actin	[15, 49]
Cholesterol depletion	Can be achieved via, e.g., nystatin and methyl‐beta‐cyclodextrin treatment to inhibit initial pit formation. Nonspecific to the CLIC/GEEC pathway as dependence on high cholesterol concentration in the local PM is common to various forms of endocytosis.	[4, 50]

The most notable and specific chemical inhibitor used to study the CLIC/GEEC pathway is LG186, a synthesized molecule that inhibits GBF1, the GEF for Arf1. Arf1 and GBF1 are necessary for CLIC/GEEC [51], and inhibition of GBF1 disrupts the ability of Arf1 to hydrolyze GTP in an efficient manner, hence disrupting the CLIC/GEEC pathway from the activation step without impacting CME or other CIE pathways. For example, Thottacherry et al., in an attempt to demonstrate the role of CLIC/GEEC in membrane tension homeostasis, used LG186 to decrease fluid‐phase endocytosis without affecting CME [7]. Alternatively, Arf1 can be depleted using RNA interference technology. Kumari and Mayor used this method to inhibit GPI‐AP, and hence formation of GEEC, while not affecting other CME or CIE pathways [34].

7‐Ketocholesterol has been identified as a main component affecting the CLIC/GEEC pathway by manipulating the packing of acyl chains on the PM [4, 38, 39, 52]. 7‐Ketocholesterol is a bioactive sterol and a major oxysterol component of oxidized low‐density lipoprotein that is produced from the oxidation of cholesterol and plays a critical role in packing the acyl chains tightly within the membrane. This oxysterol contains a sterol ring that reduces the effectiveness of its alignment with saturated acyl chains of sphingolipids in the membrane, resulting in the diminished formation of ordered membrane domains required for functional CLIC/GEEC activation sites [53]. Therefore, targeting such a component can lead to inhibiting the CLIC/GEEC pathway by preventing its initialization and GEEC formation [4, 38, 39, 52].

PICK1 can be inhibited by FSC231, which leads to a decrease in CLIC/GEEC‐mediated endocytosis in a dose‐dependent manner. Sathe et al. demonstrated that inhibition by FSC231 is specific to the CLIC/GEEC pathway as endocytic uptake of the transferrin receptor (TfR), a marker of CME, does not seem to be affected [15]. The same authors also used genetic inhibition, that is, shRNA to create stable PICK1 and IRSP53 knockdown lines to achieve results similar to chemical inhibition. Inhibition of IRSP53 also specifically leads to the complete loss of CLIC/GEEC but not CME.

Cdc42 is a key regulator of the CLIC/GEEC pathway; in the elongation step, it not only suppresses PICK1 to allow commencement of the polymerization cascade via Arp2/3 but also activates IRSp53 via binding to its CRIB domain for negative curvature enhancement. Cdc42 inhibition can be achieved by ML141, but the inhibitive effect is not specific to CLIC/GEEC [7], as Cdc42 also regulates macropinocytosis [47].

Several inhibitors are available to disrupt actin polymerization, including the inhibition of Arp2/3 via CK666, and generalized and commonly used inhibitors such as latrunculin A and jasplakinolide [15]. Similar to PM lipid domain alteration, actin polymerization broadly impacts many CME and CIE pathways; hence, its inhibition is typically not specific to CLIC/GEEC. However, Sathe et al. presented evidence of slightly stronger, dose‐dependent suppression effects of CK666 on CLIC/GEEC when compared with other pathways [15].

A lower temperature in general leads to lower metabolic rates and a decrease in endocytosis [54, 55]. However, the impact on CLIC/GEEC is more dramatic. In CHO cells, Thottacherry et al. demonstrated that CLIC/GEEC‐mediated activities appreciably decreased when temperature decreased from 37°C to 30°C and were almost completely inhibited at 25°C [7]; they also tend to recover slowly after low‐temperature incubation [4, 56]. In contrast, the dependence of CME‐based endocytosis on temperature is less dramatic, and recovery tends to be rapid after low‐temperature events. Therefore, temperature can be used to suppress CLIC/GEEC with less impact on other endocytic pathways. The inhibition is considered partially specific, with a window of higher specificity between 30°C and 37°C.

Although the involvement of GRAF1 in CLIC/GEEC is still under debate, the molecule can be inhibited by depletion via siRNA [1, 36]. In these studies, there is evidence that GRAF1 localizes and coordinates signaling of Cdc42 to facilitate GEEC internalization, and depletion of GRAF1 prohibits CLIC generation. These results therefore strongly suggest the involvement of GRAF1 in CLIC/GEEC. However, CLIC/GEEC is considered a dynamin‐independent process, while GRAF1 has been demonstrated to associate with dynamin via GRAF1's SH3 domain in affinity studies by coimmunoprecipitation in rat brain cytosol [36]. These results indicate multiple roles for this molecule in endocytic and cellular trafficking processes.

## 4 THE Cell Type Dependence of CLIC/GEEC

Different cell types harbor varying molecular mechanisms to maintain homeostasis, and not all cell lines display a robust CLIC/GEEC endocytic pathway. In Table 3, we summarize the list of common laboratory cell lines and their status regarding the ability to undergo CLIC/GEEC endocytosis.

**TABLE 3:** Cells types and evidences for presence of CLIC/GEEC pathway.

CLIC/GEEC positive	NIH 3T3 MEF [7, 57], CHO [7], AGS [7, 15], A549 [4, 58], Caco2 [4, 59], *Drosophila* [60, 61, 62], Jurkat T [45], HUVEC [63], and BHK [34]
Conflicting evidence	HEK293T [4, 64, 65], HeLa [1, 7, 14, 36, 64, 65, 66], and HepG2 [4, 9]

The most commonly used cell lines to experimentally display CLIC/GEEC are NIH 3T3 mouse embryonic fibroblasts (MEFs) and Chinese Hamster Ovary (CHO) cells. Thottacherry et al. [7] demonstrated that the MEFs exhibit a robust system for CLIC/GEEC by a combination of chemical inhibition and mechanical stimulation approaches. These studies, when paired with endocytic cargo comparison, demonstrated that CLIC/GEEC is a prevalent endocytic pathway that can be triggered by stretch‐relaxation cycling and by hypo–/isoosmotic media exposure. To differentiate between CME and CIE and evaluate the two pathways' response to stretch‐relaxation cycling, the uptake of transferrin receptors, which is a common cargo in CME, was observed in comparison to the uptake of fluid, a common indicator of CIE. To further focus on CLIC/GEEC, Thottacherry et al. used dynamin triple knockdown MEFs to inhibit all three isoforms of the endocytic scission protein, dynamin. Fluid‐phase uptake was significantly increased compared to controls while ruling out both CME and non‐CLIC/GEEC forms of CIE. Additionally, it was found that the application of both LG186 (an inhibitor of GBF1, required by CLIC/GEEC) and ML141 (an inhibitor of Cdc42, also required by CLIC/GEEC) decreased fluid‐phase uptake but had no effect on CME. These results show that CLIC/GEEC plays a significant role in the MEF stretch‐relaxation endocytic reaction. Similar experimental conditions were applied to confirm CLIC/GEEC machinery in CHO [7]. Transferrin receptor uptake was also tracked compared to folate receptors, which are a typical GPI‐AP's common for CLIC/GEEC endocytosis identification. Folate receptor uptake was significantly increased compared to controls during a cycle of cell spreading and deadhering, while transferrin receptors did not significantly change in the same cycle. This result indicates that CLIC/GEEC is not only prevalent but is also a significantly upregulated mechanism when CHO cells are exposed to stretch‐relaxation external stimuli.

Evidence‐supported presence of the CLIC/GEEC pathway for other cell types, namely, AGS [7, 15], A549 [4, 58], Caco2 [4, 59], *Drosophila* S2R+ [60], Jurkat T [45], HUVEC [63], and BHK [34] cells, is summarized in Table 3. Each cited study used similar means of typical cargo comparisons, vesicle shape and formation observations, selective inhibition of key CLIC/GEEC components, or a combination of all three to demonstrate the capability for these cell types to undergo CLIC/GEEC‐mediated endocytosis.

The evidence for other cell types, including HEK293T, HeLa, and HepG2, utilizing the CLIC/GEEC pathway, has been less conclusive and at times conflicting. For both HeLa and HEK293T cells, the occurrence of CLIC/GEEC is a debated topic [64]. For HeLa cells, studies suggest they heavily depend on dynamin and CME for uptake and lack robust CLIC/GEEC pathways [7, 14, 66], and they do not localize the required GBF1. However, studies by Nonnenmacher and Weber [64, 65] support the exhibition of the CLIC/GEEC pathway in the process of AAV2 transduction for both HEK293T and HeLa, noting functional hallmarks such as the reliance on a dynamic actin cytoskeleton and Cdc42 localization. Additionally, HeLa cells have been shown to generate tubular endocytic structures closely related to those in CLIC/GEEC pathways, further adding to the potential for mischaracterization of PM restructuring processes [1, 36]. For HepG2 cells, while one study [9] claims the presence of a robust system for CLIC/GEEC endocytosis, others argue that the observed processes appear more akin to a flotillin‐ and dynamin‐dependent pathway, which can similarly create tubular vesicles [9].

## 5 Involvement of CLIC/GEEC‐Mediated Endocytosis in DNA Transfection

The CLIC/GEEC system has been frequently indicated for fluid uptake, and characterization of CLIC/GEEC‐mediated vesicles is scarce in the literature [7, 15, 34]. In an ultrastructural study, Wang et al. observed polymorphous vesicles ranging from 50 to 300 nm in dimension in electrotransfected HUVEC cells [63], consistent with the belief that CLIC/GEEC‐mediated vesicles are similar in size to those of CME (around 100 nm) [4]. Validated and possible cargo (other than fluid) includes surface proteins such as CD44 and GPI‐AP [15], which have typical molecular weights in the range of 100 kDa, pDNA (thousands of kDa, ~100 nm) [16], and virus (AAV2, thousands of kDa) [64]. Direct observation of cargo internalization in CLIC/GEEC can be challenging, as unlike other pathways, cargos do not cluster around the forming carrier [4], hence not allowing convenient fluorescence observation. Fortunately, direct imaging of pDNA internalization by long tubular structures that are characteristic of CLIC/GEEC has indeed been observed in experimentation by Wang et al. [63] (fig. 7F,G therein). In this work, the authors utilized EP as the physical means of transfection, and CLIC/GEEC was indicated primarily in endothelial (HUVEC) cells, and with lower frequency in nonendothelial, fibroblast‐like COS7 cells. While direct observation of CLIC/GEEC involvement in these cell types is illuminating, the pertinence to applications such as cutaneous and intramuscular transfection is limited.

Prior studies on the mechanism of pDNA transfection by mechanophysical stimuli have illuminated key possibilities centered on endocytic pathways. Although EP‐mediated uptake has been described as a process where the electric field pulses create transient and reversible “pores” that permit pDNA entry [29, 67], more detailed studies have shown that uptake most likely depends on various endocytic processes [10, 12, 63, 67, 68, 69, 70, 71]. For example, Wu et al. showed that pDNA continued to gain entry to the interior of a cell for a time period much greater than the believed duration of electrically created micropores. The introduction of pharmacological inhibitors of endocytosis significantly decreased the electrotransfection efficiency (eTE) [12, 67]. Interestingly, the CLIC/GEEC pathway has also been implicated in EP‐mediated transfection, among other mechanisms, including clathrin‐mediated and caveolae‐dependent endocytosis, and Rac‐1‐dependent micropinocytosis [63, 72]. For cutaneous suction, tension and deformation are induced primarily in the skin [16]. An in vitro model was provided by Thottacherry et al. who used a matrix embedded single cell system to show that cells undergo endocytosis following the application of external stress and stretching during mechanical deformation‐mediated transfection [7]. Specifically, uptake via endocytosis was rapidly diminished while a cell experienced strain and then was quickly upregulated via the CLIC/GEEC pathway upon strain relaxation. Together, these works strongly support the hypothesis that cells have a natural reaction to external stimuli that triggers rapid and relatively long‐lasting endocytic episodes that are responsible for the uptake of pDNA when present at the cell's membrane.

Because CLIC/GEEC is activated via mechanotransduction, its role in mechanical transfection methods is naturally speculated. For in vitro studies, Geiger et al. and Hadi et al. demonstrated that various cyclic stretch methods led to enhanced gene transfection [73, 74]. However, an understanding of the MOA is absent, particularly in light of the many mechanisms by which endocytic processes are mechanically regulated [75]. For in vivo studies, Lallow et al. proposed the involvement of CLIC/GEEC in a cutaneous suction‐based transfection method, where suction is applied to the skin immediately after introducing pDNA via Mantoux injection. In the study by Lallow, the authors drew parallels with the observations of Thottacherry et al., that DNA transfection via suction occurs by CLIC/GEEC‐mediated uptake similar to the findings of Thottacherry of stretch‐mediated uptake for fluids. The reasoning was as follows: (i) Consistent with CLIC/GEEC mechanism, transfection appears to depend on relaxation postdeformation rather than deformation per se. This is seen in Figure 2 therein, where deformation application time ranges from 5 to 300 s and yet transfection effects remain quantitatively the same. The lower bound for action is merely 5 s, consistent with CLIC/GEEC as a rapid‐acting mechanism. Also consistent with the short timescale of action for CLIC/GEEC (around 90 s per [7]) is that when deformation‐relaxation is applied prior to the introduction of the pDNA, no transfection enhancement was observed [76]. It is also possible that some time is needed in order for pDNA to adhere to the membrane, such as in electroporation before intake is initiated [67, 77]. (ii) Quantitatively, the deformation thresholds are comparable. Both [7, 16] employed deformation leading to a membrane area dilation of a few percent. Particularly, [16] found that this quantitative threshold is necessary for effective in vivo transfection. These observations, together with CLIC/GEEC as a large‐capacity pathway critically mediated by the mechanically sensitive molecules including vinculin and talin, suggest CLIC/GEEC as a strong candidate for transfection in vivo in [16].

Certainly, the involvement of other pathways cannot be ruled out for these deformation‐induced DNA uptake modes. Macropinocytosis is a strong alternative candidate, as membrane stretch‐relaxation would naturally lead to ruffled regions on the membrane, resulting in internalization of the whole region by macropinocytosis. If pDNA molecules happen to be in its proximity, or adhere to the region such as in electroporation [67, 78], then they could be taken in as cargo. Indeed, Loh et al. found that a rapid decrease in membrane tension promotes membrane ruffling and triggers macropinocytosis [79]. The commonality between CLIC/GEEC and this particular case is the creation of large excess membrane area which cells recycle to establish homeostasis. In addition, they are both actin‐dependent, relying on cytoskeletal remodeling to drive membrane deformation and cargo internalization. However, these two endocytic pathways differ markedly in cargo specificity and destination, vesicle morphology, regulatory control, and physiological function (see Table 4 for a summary) [80]. Unlike the CLIC/GEEC pathway, which is selective and associated with small tubulovesicular carriers, macropinocytosis is a nonselective, actin‐driven bulk uptake mechanism that enables the internalization of large volumes of extracellular fluid, including naked pDNA and large DNA complexes. Macropinocytosis is constitutively active in certain immune cell types such as dendritic cells, macrophages, and cancer cells [47]. More commonly it is typically inducible in most cells through growth factor stimulation (e.g., colony‐stimulating factor CSF‐1, epidermal growth factor EGF, platelet‐derived growth factor, or oncogenic signaling) [81]. Although macropinocytosis provides a high‐capacity uptake route—well‐suited for large or multivalent nucleic acid complexes—the internalized cargo is often trafficked to acidic and degradative compartments, such as lysosomes. In contrast, the CLIC/GEEC pathway may offer advantages in avoiding lysosomal degradation, potentially enhancing the intracellular persistence and bioavailability of internalized pDNA [82].

**TABLE 4:** Comparison of CLIC/GEEC and macropinocytosis.

Feature	CLIC/GEEC pathway	Macropinocytosis
**Type**	Clathrin‐independent, nondynamin‐mediated	Clathrin‐independent, actin‐driven
**Main cargo**	GPI‐anchored proteins, fluid‐phase markers, certain lipids	Bulk fluid‐phase uptake, nutrients, antigens
**Vesicle size**	Small tubulovesicular structures (~100–200 nm)	Large vesicles (macropinosomes, 0.2–5 μm)
**Regulation**	Small GTPases (e.g., Cdc42, Arf1), cholesterol‐dependent, actin polymerization	Growth factor signaling, Rac1, PI3K, actin‐driven membrane ruffling
**Triggering**	Constitutive and regulated; active in many cell types under basal conditions	Usually inducible (e.g., by growth factors, oncogenic signaling), constitutively active in several cell types (e.g., macrophages, dendritic cells)
**Actin involvement**	Required for carrier formation but there is less extensive remodeling	Extensive actin rearrangement and membrane ruffling
**Dynamin Dependency**	Dynamin‐independent	Dynamin‐independent (but later stages may involve dynamin)
**Endosomal target**	GEECs (early, nonclassical endosomes)	Classical early endosomes, late endosomes/lysosomes
**Functions**	Recycling, membrane turnover, immune surveillance, GPI‐anchored protein internalization	Bulk nutrient uptake, antigen sampling, immune activation, oncogenic metabolism
**Cell types**	Active in fibroblasts, epithelial cells, dendritic cells	Prominent in dendritic cells, macrophages, tumor cells, epithelial cells

Similar to EP, deformation‐relaxation induced transfection may also likely include multiple endocytic routes, although they may be of differing and cell type‐specific frequency and prevalence [12, 63, 68]. Many endocytic pathways are mechanosensitive, with fast endocytosis and exocytosis working together to maintain constant tension homeostasis at the membrane via several different yet potentially overlapping mechanisms including the recruitment of BAR domain‐containing proteins at areas of membrane curvature (CME, CLIC/GEEC), rapid internalization in response to adhesion loss (caveola‐mediated endocytosis) or membrane wounding (calcium‐dependent endocytosis) [75]. Much more work is needed to confirm the specific MOA(s) and particularly the involvement of CLIC/GEEC, in deformation‐relaxation induced transfection, but some thoughts can be offered in the experimental design based on the current state of understanding.

## 6 Moving Forward: Experimental Designs

We conclude this minireview with some thoughts and ideas that may help establish the pertinence of CLIC/GEEC in the context of DNA transfection.

First and foremost, experiments can be designed both in vitro and in vivo to verify that the DNA transfection depends on the relaxation process, although deformation/tension increase is a necessary step to set the stage. Indirect evidence from in vivo studies by Lallow et al., as discussed above, already points to this hypothesis. This experiment could be repeated in vitro to assess the dependence of transfection efficiency on stretch‐relaxation parameters, such as the rate of tension release.

Inhibition studies can also be extended to include DNA uptake and expression to investigate the role of endocytic pathways, including CLIC/GEEC. Demonstrating dynamin independence can help rule out the involvement of CME, which can be done via both the chemical approach, using the dynamin inhibitors dynasore and dyngo 4a [83], or the biological approach, e.g., with dynamin triple knockout (TKO) MEFs [84]. If DNA uptake is dependent on CLIC/GEEC‐mediated endocytosis, and not on dynamin‐mediated mechanisms of endocytosis, then reservoir formation and DNA uptake will occur even when dynamin is inhibited or knocked out. Furthermore, validated cargos that are characteristically associated with CME, e.g., transferrin, can also be used to identify the involvement (or otherwise the absence) of CME.

Inhibition of key CLIC/GEEC‐implicated molecules, such as Arp2/3, Cdc42, and GBF1, can be performed with CK666, ML141, and LG186, respectively, following protocols established in previous studies, but with pDNA as cargo instead of fluid. As discussed in Section 3, CK666 and ML141 are nonspecific and partially specific, respectively. Furthermore, Arp2/3 is involved in both CLIC/GEEC and CME and macropinocytosis [85] and thus its inhibition effect is not restricted to CLIC/GEEC. On the other hand, although using LG186 for GBF1 inhibition is an attractive choice, it is lab synthesized and not conveniently available. An alternative option is the biological knockdown approach using ON‐TARGETplus small interfering RNAs specifically designed against GBF1 (Dharmacon), with scrambled siRNA used as a control.

Vinculin has been demonstrated to be a critical molecule that kicks off a signal cascade leading to CLIC/GEEC [7], and we have discussed its important role in mechanotransduction in the deformation‐relaxation process. However, there is no direct evidence to suggest it is involved in macropinocytosis. Therefore, targeting vinculin for inhibition could be used to differentiate CLIC/GEEC and macropinocytosis.

Temperature is another reasonable candidate to differentiate the contribution between CLIC/GEEC and other pathways including macropinocytosis. We have previously discussed that although all pathways are affected by temperature, with the physiological temperature leading to most activities for all, CLIC/GEEC seems to be most dramatically suppressed with even a slight temperature drop, e.g., to 30°C. A systematic examination of DNA transfection with deformation‐relaxation at different temperatures could therefore clarify the involvement of CLIC/GEEC.

Last but not least, direct observation can always be employed as a powerful yet lower throughput means. Imaging methods such as immunoelectron microscopy provided directly resolved structures of, e.g., tubules consistent with CLIC/GEEC and ruffled structures consistent with macropinocytosis in Wang et al. [63]. Various key molecules, such as Arp2/3, Arf1, and IRSp53 could be tagged with GFP or mCherry to facilitate direct observation [15].

In a summary, CLIC/GEEC‐mediated endocytosis is a large‐capacity and rapid‐acting mechanism that may be harnessed for in vitro and in vivo transfection needs [7, 15, 34]. This article reviewed the main molecular processes and inhibitors pertaining to the pathway, with a discussion on its relevance to various mechanophysical transfection techniques. Thus far, mechanisms of CLIC/GEEC‐mediated pathways have been extensively tackled, yet mostly with fluid and cargos other than DNA. To establish the involvement of CLIC/GEEC in DNA transfection, an integrated approach needs to be taken, where pertinent molecules and processes should be examined within the direct context of mechanotransfection.

## Author Contributions

**S.W.:** contributed to drafting the manuscript, preparing the figures, and organizing the structure of the review. **E.O.L., M.G., and C.C.R.:** contributed to drafting the manuscript, preparing the figures, and revising the content critically for intellectual accuracy. **K.K. and N.C.J.:** contributed to drafting the manuscript and preparing the figures. **D.I.S., H.L., and J.N.M.:** provided conceptual guidance, supervised the work, and critically reviewed the manuscript.

## Funding

The authors received no specific funding for this work.

## Ethics Statement

Ethical approval was not required for this review article as it does not report on original research involving human or animal subjects.

## Conflicts of Interest

The authors declare no conflicts of interest.

## Data Availability

Data sharing is not applicable to this article as no new data were created or analyzed in this study.
